# Severe Symptomatic Bradycardia Following First-Dose Oxcarbazepine in a Very Elderly Patient on Long-Term Carbamazepine: A Case Report

**DOI:** 10.7759/cureus.98837

**Published:** 2025-12-09

**Authors:** Yixin Zhang, Qinghua Yan, Jingyu Li, Guangjian Zhang

**Affiliations:** 1 Department of Pain Medicine, Yanbian University Hospital, Yanji, CHN

**Keywords:** adverse drug reaction, bradycardia, conduction abnormalities, elderly, oxcarbazepine, sodium channel blockers

## Abstract

We report a 91-year-old male patient who developed acute symptomatic bradycardia shortly after receiving his first standard dose of oxcarbazepine for trigeminal neuralgia, despite prior long-term tolerance to carbamazepine. A clear temporal association between oxcarbazepine administration and the onset of bradycardia was observed, while alternative causes were systematically excluded. Electrocardiography at baseline showed a marked intraventricular conduction delay. A subsequent ECG after drug discontinuation demonstrated an improved QRS duration, as well as new evidence of intra-atrial conduction slowing and QTc prolongation. The patient’s heart rate progressively normalized within 48 hours after oxcarbazepine withdrawal. Although the patient had tolerated high-dose carbamazepine, the occurrence of severe bradycardia after first exposure to oxcarbazepine suggests that structurally related sodium channel blockers can differ in their cardiac conduction effects. Clinicians should consider ECG monitoring when initiating oxcarbazepine in older adults or individuals with pre-existing conduction system disease or vulnerabilities.

## Introduction

Oxcarbazepine is a newer-generation antiepileptic drug whose primary mechanism of action involves the inhibition of voltage-gated sodium channels, thereby reducing neuronal excitability. Beyond its established role in epilepsy management, oxcarbazepine is widely used in the treatment of neuropathic pain, particularly trigeminal neuralgia, and has demonstrated favorable efficacy and tolerability [1,2].

Although oxcarbazepine is structurally derived from carbamazepine, it differs significantly in its metabolism. Unlike carbamazepine and other sodium-channel blockers, oxcarbazepine is not metabolized by cytochrome P450 enzymes but is instead converted to its active metabolite, the monohydroxy derivative (MHD or 10,11-dihydro-10-hydroxycarbazepine), and eliminated primarily via renal excretion, with an elimination half-life of approximately 1-3.7 hours [3,4]. This distinctive metabolic profile is believed to contribute to a reduced incidence of drug-drug interactions and certain adverse reactions [3,4].

Common adverse effects of sodium channel blockers include dizziness, somnolence, nausea, ataxia, and hyponatremia [5]. All sodium-channel-blocking antiepileptic drugs carry a potential risk of precipitating cardiac arrhythmias, particularly at higher serum concentrations [5]. However, among this drug class, reports of severe cardiac adverse events, such as symptomatic bradycardia and various forms of cardiac conduction block, remain relatively uncommon. Emerging evidence suggests that these risks may be amplified in elderly individuals or in patients receiving concomitant medications that affect cardiac conduction [6]. Although the United State Food and Drug Administration (USFDA) prescribing information acknowledges the possibility of serious cardiac reactions with oxcarbazepine [7], documented cases directly implicating the drug remain exceedingly rare.

This report describes the first documented case of severe symptomatic bradycardia with evidence of cardiac conduction abnormalities in an elderly patient following standard-dose oxcarbazepine administration. Through a detailed discussion contextualized within the existing literature, this report aims to enhance clinicians’ awareness of potential cardiac safety concerns associated with oxcarbazepine.

## Case presentation

A 91-year-old male patient was admitted with a 10-year history of right-sided trigeminal neuralgia that had markedly worsened over the preceding month. Neurological assessment identified a definite trigger point in the right mandibular division. His medical history was notable for hypertension, managed with irbesartan and nifedipine, and trigeminal neuralgia previously treated with carbamazepine at doses up to 1 g/day. Despite long-term therapy, pain control had recently become inadequate, and the patient had developed progressive neurological adverse effects, including dizziness and gait instability. On admission, physical examination revealed stable vital signs (blood pressure 167/74 mmHg).

Laboratory testing (Table 1) showed marked elevation of inflammatory markers (CRP 76.99 mg/L (reference range: 0-3 mg/L); erythrocyte sedimentation rate (ESR) 37 mm/h (reference range: 0-20 mm/h)) and elevated N-terminal pro-B-type natriuretic peptide (NT-proBNP; 2120 pg/mL; reference range: 0-125 pg/mL)), while arterial blood gas and electrolytes remained normal.

**Table 1:** Key laboratory findings on admission

Category	Test parameter	Result	Unit	Reference range
Hematology	White blood cell count	6.09	×10⁹/L	3.5–9.5
	Hemoglobin	120 ↓	g/L	130–175
	Neutrophils	71.9 ↑	%	50–70
	Lymphocytes	14.6 ↓	%	20–40
Inflammatory markers	High-sensitivity C-reactive protein (hs-CRP)	76.99 ↑	mg/L	0–3
Cardiac biomarkers	N-terminal pro-B-type natriuretic peptide (NT-proBNP)	2120 ↑	pg/mL	<125
Liver function	Albumin	32 ↓	g/L	40–55
	Gamma-glutamyl transferase (GGT)	117 ↑	U/L	10–60
	Alanine Aminotransferase (ALT)	21	U/L	9–50
	Aspartate Aminotransferase (AST)	25	U/L	15–40
Metabolic	Glucose	8.6 ↑	mmol/L	3.9–6.1
Renal function	Estimated glomerular filtration rate (eGFR)	69.77 ↓	mL/min/1.73m²	≥90
	Creatinine	84	μmol/L	57–97
	Urea	8.9 ↑	mmol/L	3.1–8.0
Urinalysis	Microalbumin	>0.15 ↑	g/L	<0.02
Electrolytes	Potassium	3.8	mmol/L	3.5–5.0
	Sodium	138	mmol/L	136–145
Arterial blood gas	pH	7.44	–	7.35–7.45
	Partial Pressure of Carbon Dioxide (pCO₂)	37	mmHg	35–45
	Partial Pressure of Oxygen (pO₂)	85	mmHg	80–100
	Bicarbonate (HCO₃⁻)	24	mmol/L	22–26
	Lactate	0.8	mmol/L	0.5–1.6

Serum albumin was mildly reduced (32 g/L; reference range: 40-55 g/L), and γ-glutamyl transferase was alone elevated (117 U/L; reference range: 10-60 U/L) with normal transaminases, a pattern typical of hepatic enzyme induction associated with long-term carbamazepine therapy. Renal assessment demonstrated age-appropriate decline in renal function, with an eGFR of 69.77 mL/min/1.73 m² and microalbuminuria, compatible with chronic kidney disease (CKD) stage G2.

Echocardiography showed preserved left ventricular ejection fraction (LVEF; 60%), concentric left ventricular hypertrophy, and a trace posterior pericardial effusion. Chest CT demonstrated mild cardiomegaly, mild bilateral pulmonary inflammation with bullae formation, and a trace right-sided pleural effusion.

**Figure 1:**
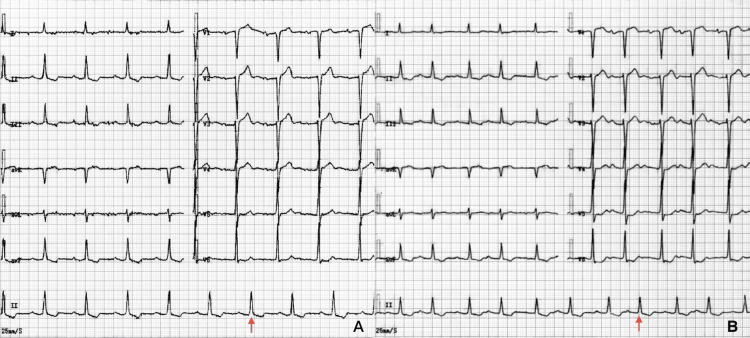
Comparative electrocardiograms at baseline and after drug withdrawal (A) Baseline electrocardiogram obtained during carbamazepine therapy, showing sinus rhythm with marked intraventricular conduction delay (QRS 176 ms) and a borderline-prolonged QTc interval (411 ms). (B) Follow-up electrocardiogram recorded 72 hours after discontinuation of sodium channel-blocking agents, demonstrating substantial improvement in ventricular conduction (QRS 120 ms; narrowing highlighted by red arrows), together with paradoxical QTc prolongation to 442 ms and a prolonged P-wave duration (64 ms) consistent with intra-atrial conduction delay.

The baseline ECG (Figure 1A) revealed sinus rhythm (68 bpm) with ST-T abnormalities in II, III, and aVF, poor R-wave progression in V2-V3, and first-degree atrioventricular block.

Automated, cycle-averaged measurements in lead II showed a P-wave duration of 32 ms, QRS duration of 176 ms, and QT/QTc of 400/411 ms.

Given the patient’s advanced age and cardiopulmonary comorbidities, a multidisciplinary evaluation was undertaken. Cardiology concluded that despite the presence of structural cardiac abnormalities and an elevated natriuretic peptide level, the patient exhibited no clinical manifestations consistent with heart failure, and continuation of the pre-existing antihypertensive regimen was recommended. The respiratory medicine team recommended initiating intravenous piperacillin-tazobactam for infection control. A repeat laboratory testing 48 hours later demonstrated a marked decline in inflammatory markers.

Because analgesia remained inadequate and carbamazepine was associated with significant neurological adverse effects, the regimen was modified. On day four of hospitalization, carbamazepine was discontinued and replaced with oxcarbazepine (300 mg every eight hours), representing the patient’s first exposure to this agent.

A temporally associated sequence of adverse cardiac events occurred shortly thereafter. On the evening following administration of the first oxcarbazepine dose (300 mg; scheduled as 300 mg every eight hours, total daily dose 900 mg) at 23:00, the patient developed chest tightness and dyspnea. Cardiac monitoring showed bradycardia (50 bpm) and oxygen desaturation to 93%, with partial improvement following supplemental oxygen. Symptoms recurred with greater severity at 01:30, when the patient developed symptomatic severe bradycardia, with heart rate decreasing to 38 bpm and blood pressure to 110/45 mmHg. Immediate intravenous atropine (0.25 mg) restored the heart rate and relieved symptoms. At 06:00, the next scheduled dose of oxcarbazepine was administered, and cardiac monitoring continued to demonstrate persistent marked bradycardia (40-45 bpm). Given the clear temporal relationship, oxcarbazepine was identified as the probable cause of drug-induced conduction disturbance and was discontinued.

**Figure 2:**
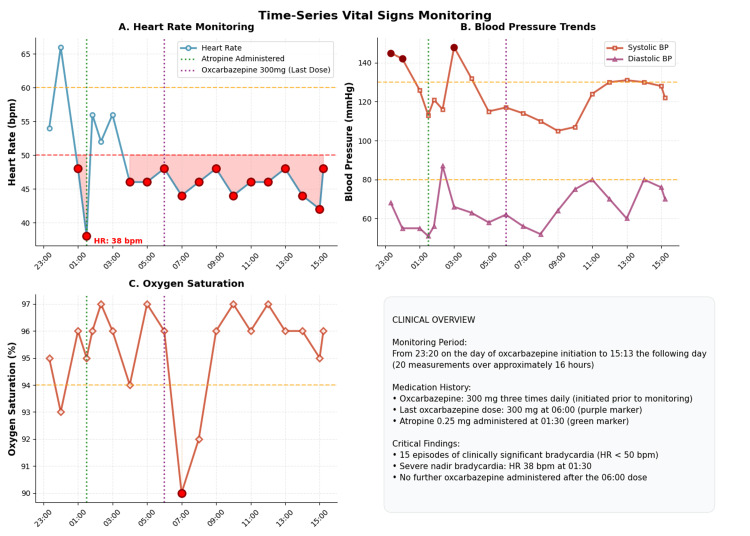
Time-series vital signs monitoring following oxcarbazepine administration Image credit: Obtained using a WEGO WGM-3012 monitor (Weigao Group, Weihai, Shandong Province, China).

The patient demonstrated time-dependent recovery following drug withdrawal. The patient's vital signs (Table 2), together with continuous time-series monitoring (Figure 2), documented progressive recovery of heart rate to 45-50 bpm within eight to nine hours, to 50-60 bpm within 24 hours, and stabilization at 70-80 bpm by 48 hours, with no further episodes of severe bradycardia.

**Table 2:** Monitoring data of the patient's vital signs Asterisk (*) indicates bradycardia (heart rate<60 bpm).

Date	Temperature (°C)	Heart rate (bpm)	Respiratory rate (bpm)
Admission (Baseline)	36.4	82	21
Hospital day 2	36.2	72	18
Hospital day 3	36.4	78	20
Day of medication change	36.1	76	17
Post-event day 1	36.5	46*	20
Post-event day 2	36.3	52*	18
Discharge day	36.6	78	19

Daily observations also revealed a stable body temperature and respiratory rate throughout the hospitalization.

A follow-up ECG obtained approximately 72 hours after complete discontinuation of sodium channel-blocking agents (Figure 1B) showed clear differential recovery within the cardiac conduction system.

In lead II, automated cycle-averaged measurements demonstrated a marked improvement in intraventricular conduction, with QRS duration narrowing from 176 ms to 120 ms (red arrows in Figure 1B). In contrast, new intra-atrial conduction slowing appeared, reflected by P-wave prolongation from 32 ms to 64 ms, along with further QTc prolongation from 411 ms to 442 ms. New-onset atrial premature complexes were present, whereas the PR interval remained essentially unchanged. Taken together, these findings indicate region-specific and nonuniform recovery across atrial, nodal, and ventricular conduction pathways following withdrawal of sodium-channel blockade. Causality was assessed using the Naranjo Adverse Drug Reaction Probability Scale [8], and the adverse event was classified as “probable” (Table 3).

**Table 3:** The assessment of oxcarbazepine-induced bradycardia via the Naranjo scale The Naranjo Adverse Drug Reaction Probability Scale is a free-to-use, non-proprietary tool for causality assessment. Adapted from Naranjo et al. [8].

Question	Response	Score
1. Are there previous conclusive reports on this reaction?	Yes	+1
2. Did the adverse event appear after the suspected drug was administered?	Yes	+2
3. Did the adverse reaction improve when the drug was discontinued or a specific antagonist was administered?	Yes	+1
4. Did the adverse event reappear when the drug was re-administered?	Do not know	0
5. Are there alternative causes(other than the drug)that could on their own have caused the reaction?	No	+2
6. Did the reaction reappear when a placebo was given?	Do not know	0
7. Was the drug detected in blood(or other fluids)in concentrations known to be toxic?	Do not know	0
8. Was the reaction more severe when the dose was increased or less severe when the dose was decreased?	Yes	+1
9. Did the patient have a similar reaction to the same or similar drugs in any previous exposure?	No	0
10. Was the adverse event confirmed by any objective evidence?	Yes	+1
Total score		8

The patient was discharged at the family's request after full disclosure of the potential risks.

## Discussion

As a sodium channel blocker, oxcarbazepine is most commonly associated with central nervous system adverse effects, such as dizziness, somnolence, and ataxia, as well as hyponatremia, hypersensitivity reactions, and hematologic abnormalities [5,9]. Cardiac adverse effects, particularly bradyarrhythmias, are rarely reported. Bradycardia is listed as a “rare adverse reaction” in the USFDA-approved prescribing information, which also notes potential cardiovascular effects, including heart failure and orthostatic hypotension [7]. A literature review identified only two prior cases of oxcarbazepine-induced sinus bradycardia in China and one case of ventricular fibrillation globally [10], underscoring the extreme rarity of severe cardiac events associated with this agent.

This case describes a 91-year-old patient who developed severe symptomatic bradycardia (nadir 38 bpm) within hours of the first oxcarbazepine dose, accompanied by acute chest tightness, dyspnea, and oxygen desaturation. The temporal proximity of drug administration and symptom onset demonstrated a classic “challenge-dechallenge” pattern. According to the Naranjo Adverse Drug Reaction Probability Scale, the causality was assessed as “probable” [8].

In considering alternative causes, the patient’s mild pulmonary infection was unlikely to precipitate such abrupt bradycardia. His inflammatory markers were already improving, and he showed no signs of systemic infection or sepsis. Furthermore, his chronic antihypertensive medications (irbesartan and nifedipine) and the antibiotic (piperacillin-tazobactam) have only weak associations with severe bradycardia, and there was no temporal link between their administration and the arrhythmia’s onset. Therefore, infection and concomitant medications were reasonably excluded as causative factors.

It is noteworthy that during the episode of bradycardia, the patient did not exhibit another common side effect of oxcarbazepine-hyponatremia or other electrolyte disturbances. This excludes hyponatremia as an intermediary factor contributing to the arrhythmia, thereby supporting the notion that the primary cause of the severe symptomatic bradycardia was the direct electrophysiological toxicity of oxcarbazepine and its active metabolite on the cardiac conduction system.

The patient had underlying cardiovascular disease, with markedly elevated NT-proBNP and echocardiographic evidence of concentric left ventricular hypertrophy, cardiomegaly, and minimal pericardial effusion. These findings are consistent with heart failure with preserved ejection fraction (HFpEF) [11]. In HFpEF, impaired ventricular compliance often renders cardiac output highly dependent on heart rate. Additionally, myocardial fibrosis is common and may involve the cardiac conduction system [12], predisposing individuals to conduction suppression through sodium channel blockade. Oxcarbazepine and its active metabolite likely exerted inhibitory effects on sinoatrial and atrioventricular nodal conduction, precipitating profound bradycardia and reducing cardiac output.

Electrocardiographic findings further support a diagnosis of drug-induced multisite conduction suppression. The baseline ECG demonstrated marked intraventricular conduction delay (QRS 176 ms), consistent with underlying structural heart disease. Seventy-two hours after the discontinuation of oxcarbazepine, the QRS interval had narrowed to 120 ms, indicating partial reversibility of the ventricular conduction impairment. This improvement suggests that the intraventricular delay was not solely attributable to pre-existing cardiac substrate but was at least partially due to sodium-channel-blocking effects. However, new intra-atrial conduction abnormalities emerged, evidenced by a significant increase in P-wave duration (32 ms to 64 ms), accompanied by additional QTc prolongation (411 ms to 442 ms). Frequent atrial premature complexes observed prior to discharge further suggested that the atrial electrical instability was likely related to oxcarbazepine exposure. Together, these ECG features reflect heterogeneous and region-specific vulnerability across the atrial myocardium, nodal tissue, and ventricular conduction pathways, possibly indicating differential sensitivity to sodium channel-blocking agents. Sequential improvement in QRS duration, contrasted with the persistence of atrial conduction delays, suggests differential susceptibility among components of the cardiac conduction system.

Multiple studies have suggested that antiepileptic drugs, particularly sodium channel blockers, may serve as independent risk factors for sudden cardiac death, even among non-epileptic individuals [13]. Most antiepileptic drugs primarily target voltage-gated sodium channels but possess off-target activity, including calcium channel inhibition [3,14]. Voltage-gated sodium and calcium channels are expressed in both cardiac and neural tissues, with distinct subtype distribution patterns across these tissues [15]. Due to the non-absolute binding affinity of brain channel subtypes, sodium channel-blocking antiepileptic drugs may affect cardiac function either directly through interaction with cardiac tissue or indirectly by modulating the central autonomic nervous system, potentially leading to cardiac dysfunction and arrhythmias [15].

All sodium channel blockers carry arrhythmogenic potential, particularly at high concentrations [5]. Evidence increasingly supports a class-wide effect of cardiac conduction suppression. A large-scale study, involving 30,077 subjects, found a conduction delay prevalence of 36.1% with carbamazepine, 45.5% with phenytoin, and 54.7% with lamotrigine [6], demonstrating that sodium channel blockade is clinically meaningful across multiple agents in this pharmacologic class. However, in this study [6], data related to oxcarbazepine were limited to only one case, preventing statistical significance from being reached.

Although the patient had tolerated high-dose carbamazepine, the development of severe bradycardia upon first exposure to oxcarbazepine may reflect a functional divergence in cardiac safety profiles between these structurally related agents. Oxcarbazepine has been shown to exert sodium channel blockade at relatively low concentrations, raising the possibility of higher affinity for cardiac sodium channels. Additionally, its distinct calcium channel modulation profile-targeting N-, P-, and R-type currents-differs from carbamazepine’s primary activity on L-type calcium channels [3,16,17]. This differential ion-channel modulation may contribute to enhanced nodal and atrial conduction suppression by oxcarbazepine. These mechanistic considerations are consistent with a possible lack of cross-tolerance at the cardiac level, whereby long-term carbamazepine exposure does not confer protection against conduction suppression induced by oxcarbazepine.

Oxcarbazepine is primarily metabolized to its active metabolite MHD by ketoreductases, followed by renal elimination. In contrast, carbamazepine is metabolized mainly by hepatic cytochrome P450 enzymes [18]. In elderly patients with age-related reductions in renal clearance, an underlying state of renal impairment may facilitate the accumulation of MHD [4]. In this case, the patient exhibited several vulnerability factors reflecting diminished physiological reserve even before the switch to oxcarbazepine. From a hepatic perspective, long-term carbamazepine therapy had already exerted a measurable impact on hepatic enzymatic activity. His laboratory findings demonstrated an isolated and markedly elevated γ-glutamyl transferase (GGT) level with otherwise normal transaminases, a pattern characteristic of drug-induced hepatic enzyme induction rather than acute hepatocellular injury. This pre-existing inductive state, when considered alongside his age-related decline in renal function (CKD G2 with microalbuminuria), suggests a compromised overall capacity to metabolize and eliminate drugs. Furthermore, residual carbamazepine or its metabolites may have contributed to a cumulative inhibitory effect on cardiac sodium channels, creating a synergistic burden that precipitated the profound bradycardia observed in this case.

Although oxcarbazepine is generally considered to have a favorable cardiac safety profile [19], the paucity of documented adverse cardiac events likely reflects underreporting or limited awareness rather than an absence of risk. Recent FDA warnings regarding lamotrigine and updated recommendations from the International League Against Epilepsy and the American Epilepsy Society highlight the need to reassess cardiac safety for all sodium-channel-blocking antiepileptic drugs [20]. It is now advised to perform routine ECG screening and enhanced cardiac monitoring for patients ≥60 years old or those with underlying arrhythmia risk factors when using these medications [20].

Limitations of this report include the absence of invasive electrophysiological testing, lack of serum MHD concentration measurements, incomplete assessment of autonomic status at the time of bradycardia, and lack of ECG capture during the most severe episode. Nonetheless, the abrupt onset of profound symptomatic bradycardia immediately following the first oxcarbazepine dose, rapid and sustained improvement after drug discontinuation despite unchanged concomitant medications, and the temporal evolution of ECG abnormalities collectively implicate oxcarbazepine as the primary causative factor. No alternative explanation adequately accounts for the observed electrophysiological deterioration.

## Conclusions

This case report describes the complete clinical course of severe symptomatic bradycardia in an elderly patient who had previously tolerated carbamazepine but developed acute conduction abnormalities following first-time exposure to oxcarbazepine. These findings provide supportive clinical evidence of oxcarbazepine-induced cardiac conduction suppression, addressing an important gap in the current safety profile of this sodium channel-blocking antiepileptic drug.

Although oxcarbazepine is generally regarded as possessing a favorable cardiac safety profile, this case highlights its potential for serious cardiac events and suggests a possible lack of cross-tolerance with carbamazepine at the level of cardiac conduction. The uniqueness of this case lies in the uncommon nature of oxcarbazepine-related bradycardia and the clinically relevant observation that prior long-term tolerance to carbamazepine does not necessarily predict cardiac safety after switching to oxcarbazepine. It is imperative to exercise heightened vigilance, including baseline and follow-up ECG monitoring, when prescribing oxcarbazepine to elderly patients or those with cardiac risk factors, regardless of prior carbamazepine tolerance.
